# EFFECTS OF FUNCTIONAL BRAIN NETWORKS AND WHITE MATTER DISEASE ON MOBILITY OF OLDER ADULTS IN AN EXERCISE INTERVENTION

**DOI:** 10.1093/geroni/igz038.3132

**Published:** 2019-11-08

**Authors:** Blake R Neyland, Robert Kraft, Mary Lyles, Stephen Kritchevsky, Paul J Laurienti, Barbara M Nicklas, Christina Hugenschmidt

**Affiliations:** Wake Forest School of Medicine, Winston-Salem, North Carolina, United States

## Abstract

Declining mobility is associated with increased accumulation of white matter hyperintensities (WMH). However, a high WMH burden is not always accompanied by impaired mobility. Our previous work demonstrates that some variance in mobility may be explained by brain network connectivity. Here, we extended this work by measuring WMHs and brain networks in older adults participating in a lifestyle intervention. The Short Physical Performance Battery (SPPB) and resting state functional magnetic resonance imaging (fMRI) were collected before and after a 5-month caloric restriction plus aerobic exercise intervention in 57 obese, sedentary adults aged 65-78. Participants were categorized based on median splits of baseline SPPB scores and WMH burden: Expected Healthy (EH: low WMH, SPPB≥11, n=16), Expected Impaired (EI: high WMH, SPPB≤10, n=17), Unexpected Healthy (UH: high WMH, SPPB≥11, n=12), and Unexpected Impaired (UI: low WMH, SPPB≤10, n=12). Graph theory-based methods were used to characterize brain networks and compare the four groups. At baseline, the somatomotor cortex community structure (SMC-CS) was less consistent in EI (p=0.05) and UI (p=0.23) compared to EH. The EI (mean=1.25, p=0.003) and UI (mean=1.57, p=0.001) significantly improved their SPPB scores following the intervention. Although both groups had equivalent SPPB scores, SMC-CS was less consistent in the UH than EH (p=0.16). However, UH displayed a significant (p=0.004) increase in second-order connections to the precuneus compared to EH. These data suggest that studying brain networks could improve the understanding of the development of mobility disability and the CNS contributions to mobility independent of white matter disease.

